# Retrievability of Fractured Abutment Screws in Dental Implants Using Three Removal Techniques: An In Vitro Pilot Study

**DOI:** 10.3390/jfb17020085

**Published:** 2026-02-09

**Authors:** Ming-Dih Jeng, Tzu-Yun Huang, Amber Yeh Jeng

**Affiliations:** 1 Department of Dentistry, Far Eastern Memorial Hospital, New Taipei City 220216, Taiwan; mdjenq@ms12.hinet.net (M.-D.J.); a98098090y@gmail.com (T.-Y.H.); 2 Department of Dentistry, Kaohsiung Medical University, Kaohsiung City 807378, Taiwan

**Keywords:** screw fracture, retrievability, fissure bur, screw removal kit, ultrasonic scaler, implant connection deformation

## Abstract

**Introduction**: The fracturing of abutment screws is a recurrent technical complication in implant-supported prostheses that may compromise prosthetic maintenance. Although multiple retrieval approaches have been described, comparative data under controlled experimental conditions remain limited. **Materials and Methods**: This in vitro pilot study evaluated the retrievability of fractured abutment screws when using three commonly applied instruments: an ultrasonic scaler, a fissure bur, and a screw removal kit. Eighteen implants from a single implant system were embedded in epoxy resin, and abutment screws were fractured under clockwise monotonic torque either with (w/A) or without (w/oA) abutments (*n*= 3 per retrieval method). Retrieval success and procedure time were recorded. Scanning electron microscopy (SEM) was performed to qualitatively assess deformation of the implant internal hex and screw thread morphology. **Results**: Fracture torque values were higher in specimens fractured with abutments compared with those without abutments. Fractures induced without abutments appeared to extend deeper within the screw channel, engaging a greater number of internal threads. In this pilot study, a shorter retrieval time was observed with the screw removal kit and fissure bur compared with the ultrasonic scaler, although retrieval outcomes varied between specimens. SEM observations suggested differing patterns of internal hex deformation between the retrieval techniques. **Conclusions**: Within the limitations of this in vitro pilot study, different retrieval approaches demonstrated characteristic mechanical behaviors and deformation patterns in the implant internal connection. These preliminary findings provide descriptive insight into the retrievability of fractured screws and may serve as a basis for future studies with larger sample sizes and clinically relevant fracture models.

## 1. Introduction

Dental implants have been widely used to replace missing teeth with predictable long-term outcomes; however, various complications continue to be reported in clinical practice, including biological and mechanical problems [1,2]. Among the mechanical complications, abutment screw fracture represents a challenging issue that may compromise prosthetic function and maintenance. Recent biomechanical evidence has shown that the mechanical stability and stress distribution of implant systems are strongly influenced by the configuration of the implant–abutment–screw interface, underscoring the importance of preserving the integrity of the internal connection during secondary interventions [3]. Numerous previous studies have examined the mechanisms of screw fracture and loosening, as well as various approaches for retrieving fractured screw fragments; nevertheless, most investigations have focused on clinical feasibility rather than controlled mechanical comparisons.

Beyond being classified as a technical complication, the retrievability of fractured abutment screws can have clinically relevant implications in specific treatment contexts. In full-arch and immediate loading rehabilitations, screw-related complications are often part of long-term maintenance demands, highlighting the importance of preserving implant–abutment interface integrity during long-term prosthetic maintenance. Previous long- and mid-term studies have demonstrated that such complications may influence maintenance demands and prosthetic manageability over time, even when implant survival remains high. In these clinical scenarios, the ability to retrieve fractured screws while preserving the structural integrity of the implant–abutment connection is therefore clinically relevant [4,5].

Several studies have suggested that the screw fracture process may exhibit typical fatigue-related features. Plastic deformation of the screw surface can occur during tightening, particularly when high insertion torque is applied. Such deformation may increase the stress concentration and has been proposed to facilitate hydrogen absorption, which could contribute to delayed brittle fracture under certain conditions [6]. However, these mechanisms remain multifactorial and context-dependent.

In addition, previous studies have shown that even in precisely machined screws, the tightening process is accompanied by a settling effect, in which contact points undergo plastic deformation and material flow. Repeated tightening–loosening cycles may further lead to desquamation and damage to the thread surface, resulting in a reduction in preload and a substantially increased risk of screw loosening [7,8].

Surface alterations and deformations of screw threads have been reported following torque application and abutment loosening. Deformation typically occurs at the convexities of the threads after tightening, whereas cyclic loading may induce fatigue cracks at the concavities. As a result, reusing a loosened screw with accumulated deformation has been associated with an increased risk of subsequent fracture [9].

Recent reviews have highlighted that technical complications such as screw loosening and fracture remain persistent challenges in implant dentistry and may influence long-term prosthetic maintenance [10]. Reported incidence rates of screw-related technical complications vary widely, reflecting differences in the prosthesis type, implant system design, observation period, and definitions of complications. In contrast, implant fracture itself has been reported at much lower rates, ranging from approximately 0.16% to 1.5% of cases [11,12,13]. Although advances in implant design and tightening protocols have been introduced, comparative evidence regarding fractured screw retrieval techniques under controlled experimental conditions remains limited. As a result, the clinical management of fractured abutment screws is often influenced by operator experience.

Previous studies have evaluated various approaches for fractured screw retrieval. For example, Agustín-Panadero et al. reported that the use of a dedicated screw removal kit without a guide cylinder demonstrated favorable retrieval performance compared with explorer probes or ultrasonic tips [14]. Other studies have proposed alternative screw designs, such as hollow abutment screws, which may facilitate removal without significantly compromising mechanical performance [15]. However, these investigations primarily focused on clinical feasibility or specific design modifications, with limited emphasis on controlled mechanical comparisons and implant internal connection deformation evaluations.

From a mechanical–technical perspective, fractured screw retrieval techniques can be categorized according to distinct operative principles, including vibrational loosening using ultrasonic devices, mechanical engagement with reverse rotation using screw removal kits, and material modification through drilling to facilitate force transmission [14,16]. The effectiveness of these approaches and the risk of internal damage are influenced by the implant connection geometry and fracture depth, as the connection design determines the stress distribution and fracture location; for example, internal connections—such as conical (Morse taper) designs—enhance mechanical stability but often result in deeper fracture sites and limited access, thereby increasing retrieval difficulty and the need to balance efficiency with preservation of internal threads [7,16,17].

Although no standardized method has been established for screw removal to date, various techniques have been reported for this purpose, such as the use of artery forceps, reverse low-speed burs, ultrasonic scalers, and screw removal kits. In this study, abutment screws were manually fractured within the implant under controlled experimental conditions with (w/A) and without (w/oA) abutment engagement, following which retrieval was attempted using an ultrasonic scaler, a fissure bur, or a screw removal kit. The objective was to evaluate the efficiency, advantages, and disadvantages of these three methods for removing fractured screws.

Accordingly, the novelty of the present study does not lie in the comparison of retrieval methods alone, but in the integrated evaluation of (1) fracture depth characteristics with and without abutment engagement, (2) retrieval behaviors and procedure times under controlled in vitro conditions, and (3) patterns of internal implant connection deformation assessed via scanning electron microscopy. By addressing these aspects within a single experimental framework, this study aims to fill a specific mechanical–technical knowledge gap related to the integrity of the implant internal connection.

## 2. Materials and Methods

### 2.1. Sample Preparation

**Figure 1:**
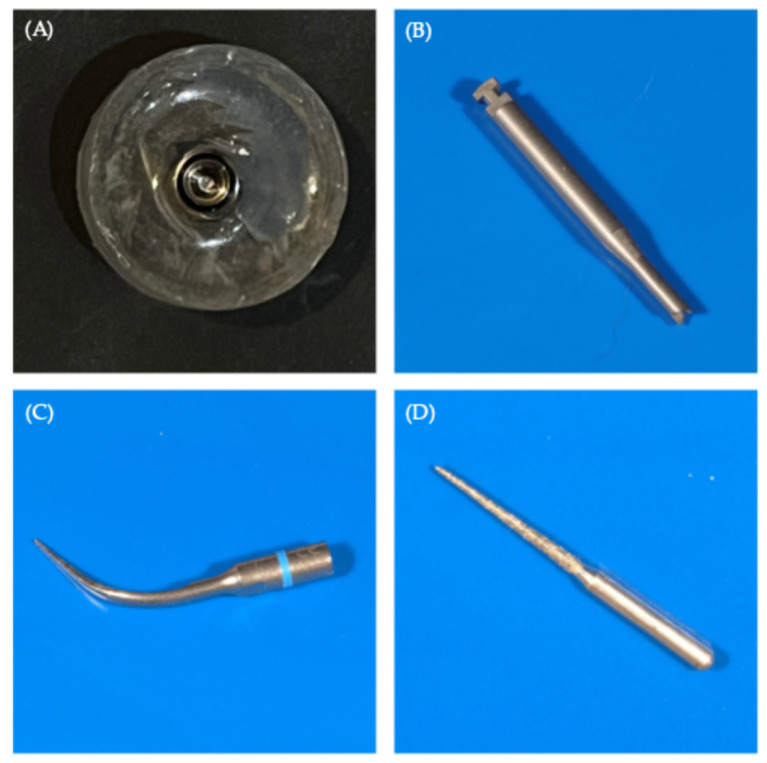
Epoxy resin embedding and materials of removal of fractured screws. (**A**) Top view of the dental implant embedded in epoxy resin, with the arrow pointing to the fractured screw, (**B**) screw removal kit with 1.5 mm tips under microscope view at 4× magnification, (**C**) ultrasonic scaling tip under microscope view at 4× magnification, and (**D**) fissure bur under microscope view at 4× magnification.

Eighteen commercially available dental implants (Ø3.5 × 13 mm, lot number: IF3513, Grade IV Titanium; Ideoss Dental Inc., Taipei, Taiwan) were used in this study. To standardize the resin-implant geometric relationship, each implant was positioned at the center of a cylindrical plastic mold (internal diameter of 16 mm), fixed to the base of the mold, and aligned coaxially with the long axis of the mold. The implants were embedded in epoxy resin (Cheng Yi Chemical Co., Taipei, Taiwan) blocks, leaving 2 mm of the implant shoulder exposed to allow access to the abutment and screw (Figure 1A).

The interface between the implant and abutment is an internal connection featuring the combination of a 1.7 mm morse taper and a 1.3 mm hex. The abutment (lot number: AA3515005, Grade V Titanium) with an external hex was chosen, and a separated abutment screw (lot number: AS1484, Grade V Titanium) was inserted with a driver to apply a clamping force and secure the abutment and implant complex; the manufacturer-recommended tightening torque is 25 N·cm, which served as the reference clinical tightening value prior to fracture induction.

To induce artificial screw fracture, the 18 samples were divided into two groups: with abutment (w/A) and without abutment (w/oA). All samples were mounted in a digital torque measurement device (Tohnichi BTG 60Z, Tohnichi Mfg. Co., Tokyo, Japan), and torque was applied continuously through the device. Fracture was defined as an abrupt torque drop exceeding 20 N·cm, and the peak torque value immediately prior to fracture was recorded. In the w/A group, the abutment was placed on the implant and the screw was tightened under torque control until fracture occurred. In the w/oA group, the screw was initially gently inserted and directly rotated into the implant without abutment engagement until fracture occurred, and the torque was measured using the same torque gauge device.

Fracture induction was performed using monotonic torque to failure. This loading condition does not replicate fatigue-related fracture mechanisms commonly observed in clinical settings and was therefore considered a methodological limitation of this in vitro model.

### 2.2. Removal of Fractured Screws

The fractured remaining parts of the abutment screws were subjected to retrieval using one of the following instruments. First, the screw removal kit, which contained a serrated end to provide high frictional force on the screw fragment, was purchased from Cosmos Dental Technology Co. (Guangdong, China) and was driven by an implant handpiece (NSK, Surgic Pro, Tochigi, Japan). Second, a P5 Newtron Satelec scaler tip #1S (Aceton, Mérignac, France), which applied vibrating motion to the screw. Third, a fissure bur (lot number: 217mF, Dadong, Taiwan) engaged in a high-speed turbine (NSK, Tochigi, Japan).

Samples with artificially fractured screws were assigned to three removal methods—ultrasonic scaler, high-speed fissure bur, and screw removal kit—with 3 samples per group. All procedures were performed by a single operator (M.J.) to minimize operator-related variability, under a microscope (M320 MultiFoc Objective, Leica, Singapore). Samples were randomly assigned to the corresponding removal method and recorded on video.

1. Screw removal kits (Figure 1B): The tool was lightly placed on the fractured screw’s surface and rotated at 50–100 rpm with the maximum torque setting of the device (80 N·cm) using an implant handpiece.
2. Ultrasonic scalers (Figure 1C): The tip of the ultrasonic scaler was placed on the top of the screw, and the highest power setting of the device was applied with irrigation and manual counterclockwise rotation to gradually unlock the fractured screw until the fractured screw was removed.
3. High-speed fissure burs (Figure 1D): The bur was rotated clockwise and lightly touched onto the side of the fractured screw to make the screw fragment rotate counterclockwise.

Two outcome parameters were evaluated for each removal method: (1) successful complete removal of the screw fragment from the screw channel, and (2) removal time, defined as the duration from initiation of instrument operation until the fractured fragment was entirely extracted from the implant screw channel.

### 2.3. Evaluation of Removal Efficiency and Surface Alterations

After the removal procedure, all specimens were examined using a scanning electron microscope (Tabletop Microscope TM3000, Hitachi, Tokyo, Japan) to assess surface alterations of the implant internal connection, including the internal hex and taper regions. The SEM analysis was qualitative in nature and performed under consistent imaging conditions. An unused original implant from the same system was examined as a reference control for comparison.

### 2.4. Statistical Methods

Numerical data were analyzed using SigmaPlot, with descriptive statistics presented as means ± standard deviations. Inferential statistical tests (ANOVA with Tukey’s post hoc test and chi-square test) were applied in an exploratory manner, due to the limited sample size (*n* = 3 per group). Accordingly, the presented statistical results should be interpreted with caution. A nominal significance level of *p* < 0.05 was used for exploratory comparison.

## 3. Results

**Figure 2:**
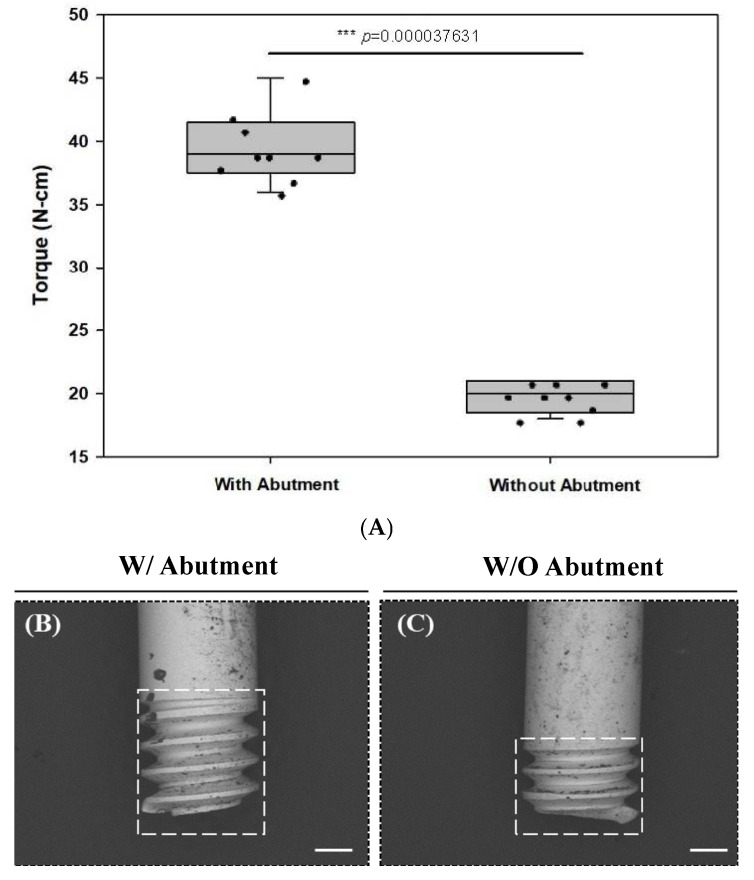
Torque measurement of artificially fractured screws with and without abutments: (**A**) Torque required for screw fracture with abutment (w/abutment) and without abutment (w/o abutment) (*n* = 9, *** *p* < 0.001), (**B**) SEM image of the screw artificially fractured with an abutment (scale bar: 400 µm), and (**C**) SEM image of the screw artificially fractured without an abutment (scale bar: 400 µm).

In the presence of an abutment (w/A), the mean screw fracture torque was 39.6 ± 2.58 N·cm, whereas without an abutment (w/oA) it was 19.8 ± 1.13 N·cm. The torque required to fracture the screw differed significantly between the two groups (Figure 2A, *p* < 0.001). The fracture characteristics also differed between groups, primarily regarding fracture location and fragment engagement within the implant. Therefore, the fractured screw fragments were further examined using scanning electron microscopy.

The depth of the screw fractures was analyzed via SEM. As the fragment in the w/oA group could not be removed, only the shank part of the screw was compared. Based on representative SEM observations, the fracture location in the w/oA group appeared to be positioned approximately 400 µm closer to the coronal aspect of the screw compared with that in the w/A group (Figure 2B,C). In the w/oA specimens, a greater number of threads remained engaged within the implant chamber.

The nine samples with fractured screws inside abutments were randomly assigned to one of three removal methods: ultrasonic scaler, fissure bur, or screw removal kit (three samples per group). After grouping, the fracture torque was further evaluated in each group: the values for the ultrasonic scaler, fissure bur, and screw removal kit groups were 40.66 ± 1.24, 40.33 ± 3.39, and 39.33 ± 1.25 N·cm, respectively, with no statistically significant differences between the groups.

The retrieval time and success outcomes for each method are illustrated in Table 1. In the ultrasonic scaler group, one specimen could not be retrieved despite subsequent attempts using a screw removal kit and a fissure bur. The remaining two specimens were retrieved within 55 and 110 s, respectively. In the fissure bur group, all three specimens were successfully retrieved, with removal times of 24, 26, and 48 s. In the screw removal kit group, all three specimens were successfully retrieved, with removal times of 23, 18 and 42 s. The removal times are summarized as mean ± standard deviation to describe the trends observed among the retrieval methods.

Overall, mean removal times (± SD) were 27.6 ± 10.3 s for the screw removal kit, 82.5 ± 27.5 s for the ultrasonic scaler (based on two successful specimens), and 32.3 ± 11.1 s for the fissure bur.

In the w/oA group, retrieval attempts were performed using all three methods. The screw removal kit and ultrasonic scaler were extended to durations approximately twice those applied in the w/A group (60 s for the screw removal kit and 3 min for the ultrasonic scaler); however, no observable movement of the fractured fragment was detected. Fissure bur application was not feasible due to the absence of an exposed lateral surface of the fractured fragment. Consequently, none of the w/oA specimens could be retrieved under the present experimental conditions.

**Figure 3:**
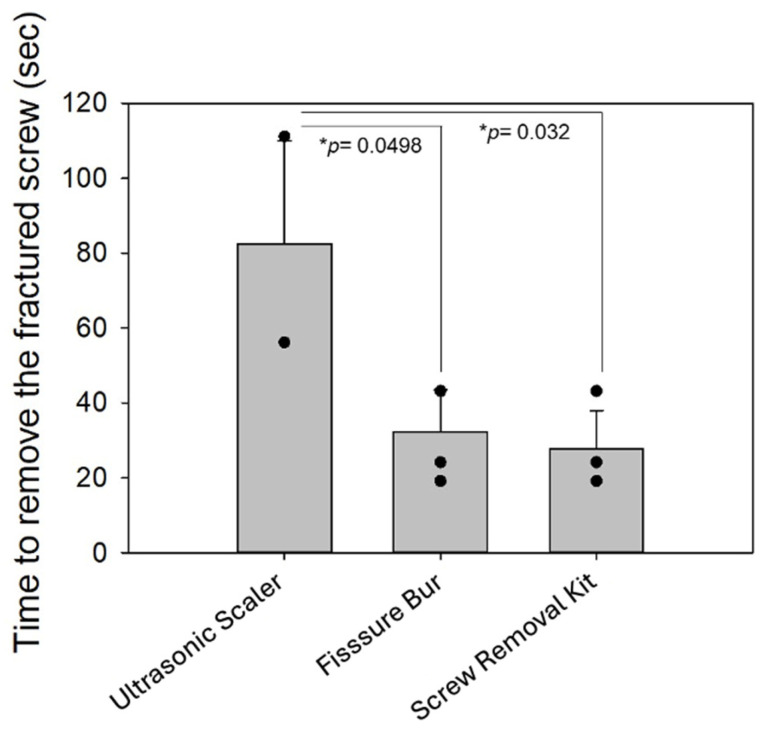
The time required to remove the screws with three methods: ultrasonic scaler, fissure bur, and screw removal kit. * *p*-value < 0.05.

Although exploratory statistical comparisons were performed, the results should be interpreted with caution due to the limited sample size (*n* = 3 per group). Under the present in vitro conditions, retrieval times and success rates varied between the tested methods. The ultrasonic scaler was associated with greater variability in retrieval time and one unsuccessful retrieval, whereas the fissure bur and screw removal kit were associated with shorter retrieval times in the successfully retrieved specimens (Figure 3).

**Figure 4:**
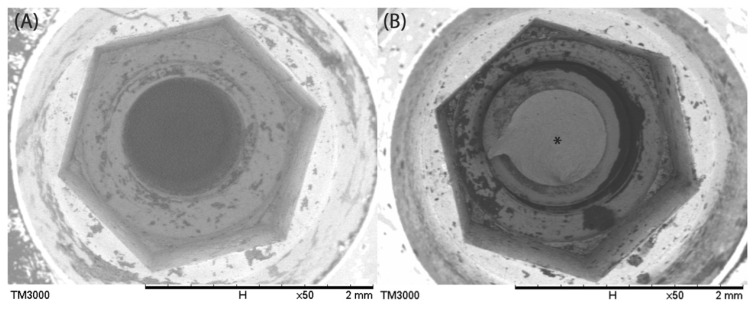
SEM images showing the internal hex connection: (**A**) Control and (**B**) implant (w/A) before removal of fractured screw (*), which extends out of the screw channel within the internal hex.

SEM images of the internal hexagonal and taper parts of the original implant (control, Figure 4A) and the screw fractured with an abutment (w/A, Figure 4B) illustrated the position of the fracture screw. The screw fragment remained below the entrance of the internal hex, shifted toward one side of the channel, and extended into the trench.

**Figure 5:**
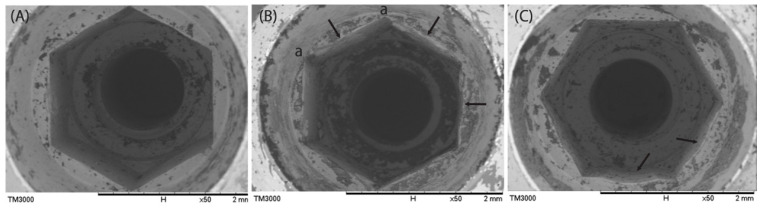
SEM images of implants after removal of fractured screws via three different methods: (**A**) Ultrasonic scaler, (**B**) fissure bur, and (**C**) screw removal kit. Arrows point to damaged sides and letter “a” indicates the jeopardized angle of the hex.

Implants with abutment subjected to the three different screw removal methods (i.e., ultrasonic scaler, fissure bur, and screw removal kit) were compared to evaluate the damage caused by each removal technique. SEM examination revealed surface alterations of the internal hex and taper regions in implants subjected to fissure bur and screw removal kit retrieval. In contrast, minimal observable surface alteration was noted in specimens retrieved using the ultrasonic scaler (Figure 5). Compared with the control group, the internal compartment in the ultrasonic scaler group did not exhibit significant deformation (Figure 5A). In the fissure bur group (Figure 5B), deformation of the internal hex was observed (arrows point to the deformed areas in the figure). In the removal kit group (Figure 5C), internal thread deformation was also observed but was less severe than that in the fissure bur group. 

In summary, the results demonstrated differences in fracture characteristics, retrieval behavior, and internal surface morphology between the tested retrieval techniques under the present in vitro conditions, with each method associated with a distinct pattern of retrieval time, success rate, and observable implant surface alteration.

## 4. Discussion

At the end of the last century, Esposito defined late failure as implant removal after prosthesis delivery [18] and Sakka described the causes of late failure, including peri-implantitis, excessive occlusal loading, and inadequate prosthetic design [19]. These authors considered that screw loosening and fracturing were associated with the occlusal force and prosthetic pattern.

The philosophy of implant selection in this study was that if a tool can effectively remove a screw from a narrow channel, it should be capable of entering a wider access hole and retrieving a screw fragment. Therefore, the implant of smallest diameter in this system with a minimal access channel was used.

According to a previous study, a solid abutment endured a lower torque strength than a screw–abutment combination; however, the former left a longer fragment in the implant chamber than the latter [20]. Therefore, this study designed a screw–abutment complex rather than a solid abutment, as retrieval techniques that are applicable for removing short fragments should also be applicable to long fragments. Furthermore, the previous study also reported the solid abutment lost less torque and presented a higher endurance limit than a screw-retained abutment, which is more vulnerable to occlusal force and is more likely to fracture in a clinical situation.

The incidence of abutment screw fractures has been reported to be around 2% in clinical practice [1]. Additionally, the rate of screw loosening over a 5-year period was reported to range from 3.9% to 26.2% in studies published before 2000, but decreased to between 3.1% and 10.8% in studies published after 2000 [2,21]; a similar result was also noted regarding the occurrence of screw loosening, which ranged from 25% for single implants in an earlier study to 8% in more recent research [22]. The above statistical outcomes likely reflect advancements in implant and screw designs and tightening protocols [7,23].

For instance, in a previous finite element study analyzing the effect of implant wall thickness on the stress of the abutment screw in an implant system designed for internal connection, the author found that a thinner implant wall led to higher strain on the abutment screw [24]. Beyond a fixture wall thickness of 0.45 mm, the stress of the abutment screw dramatically increased; meanwhile, the ideal implant strength protected the inner screw.

Most currently used implant designs present an internal taper connection, rather than an external hex; the former design lessens the static micro gap [25], further reducing this micro gap after cycle loading and abolishing micromovement of the abutment [26,27,28]. Consequently, when the conical seal featured a cold solder situation at the implant–abutment interface, the incidence of screw damage or loosening decreased [21,29]. With the increasing number of studies focused on implant design over the past decade, the amount of screw complications has consequently decreased. 

Notably, fractured screws tightened with an abutment exhibit two special features: they remain loosely positioned, with a few threads projecting outside the screw channel, allowing a high-speed bur to contact the lateral side of the fragment and unscrew it. By contrast, screws that fracture without these two characteristics may require a removal kit.

A clamping force between the abutment and implant is applied when a screw fractures, leaving a loose fragment inside the implant and enabling the screw to be removed by a kit or a bur. However, screws without an abutment often become tightly lodged within the screw hole and cannot be removed by any method. In this study, the scaler, removal kit, and high-speed bur were only effective at applying low torque to rotate loose screws (i.e., in the abutment case). 

From a mechanical–experimental standpoint, the present findings suggest that the retrievability of a fractured screw may vary depending on the fragment’s condition and engagement, rather than supporting a single universally effective technique [5]. In this experimental model, when the fractured screw fragment remained loose and partially exposed within the implant connection, mechanical approaches—particularly the screw removal kit and fissure bur—consistently allowed for faster retrieval. Nevertheless, SEM observations revealed varying degrees of internal thread deformation, which may be related to lateral contact, high-speed rotational slip, and uncontrolled engagement of the fissure bur with internal hex corners, which reflects mechanical interactions between the retrieval instruments and the implant’s internal geometry. In contrast, the ultrasonic scaler showed less predictable removal performance but minimal damage to the implant connection. Within the context of this in vitro model, this trade-off highlights differences in mechanical interaction between retrieval techniques, rather than indicating specific clinical indications.

Among the screws tested with abutments, only one sample in the ultrasonic scaler group failed to be removed. Although the chi-square test did not show statistical significance, the results suggest the variable performance of ultrasonic scalers in this experimental model. Furthermore, once a fractured screw is subjected to ultrasonic vibration, subsequent attempts with a removal kit or a high-speed bur may also fail. Therefore, any potential influence of ultrasonic vibration on subsequent retrieval attempts remains speculative and cannot be confirmed based on the present data.

Lastly, it is important to emphasize that this study employed a single implant system featuring a specific internal taper and hex connection, and the findings should not be generalized to implant systems with different abutment retention mechanisms. In addition, the use of a monotonic torque-induced fracture model, epoxy resin embedding, small sample size, qualitative SEM assessment without quantification, and operator-dependent performances represent important limitations of this pilot study.

Looking forward, data-driven and AI-assisted risk models may offer additional tools for exploring technical complication risks and long-term maintenance demands by analyzing complex relationships between biological and mechanical variables. With the accumulation of longitudinal clinical data, such approaches could support stratified risk assessments and aid in identifying situations where more conservative retrieval strategies may be preferable to limit mechanical damage. However, the clinical utility of these models remains exploratory, and their integration into implant maintenance decision making will require further validation through prospective studies [30].

## 5. Conclusions

Within the limitations of this in vitro pilot study, screw removal kits and fissure burs were associated with shorter retrieval times but greater deformation of the implant internal geometry, whereas ultrasonic scalers resulted in minimal observable damage but demonstrated less predictable retrieval success. These findings suggest that the retrieval of fractured screws is affected by a trade-off between retrieval efficiency and preservation of the implant internal connection. However, the results of this study should be interpreted cautiously, as they may not be generalizable to other implant systems, screw designs, or fatigue-related fracture scenarios. Further investigations using larger sample sizes, different implant connection designs, and clinically relevant fracture models are required to validate these preliminary observations.

## Figures and Tables

**Table 1:** Individual retrieval times (seconds) and outcomes for each removal method. N/A referred unsuccessful removal.

	Sample 1	Sample 2	Sample 3
Screw removal kit	23	18	42
Ultrasonic tip	55	110	N/A
Fissure bur	24	26	48

## Data Availability

The raw data supporting the conclusions of this article will be made available by the authors on request.

## Abbreviations

The following abbreviations are used in this manuscript:

SEM	Scanning electron microscope
SD	Standard deviation
w/A	With abutment
w/oA	Without abutment
